# Alpha-1 antitrypsin gene polymorphism in Chronic Obstructive Pulmonary Disease (COPD)

**DOI:** 10.1590/S1415-47572009005000107

**Published:** 2010-03-01

**Authors:** Sabri Denden, Amel Haj Khelil, Jalel Knani, Ramzi Lakhdar, Pascale Perrin, Gérard Lefranc, Jemni Ben Chibani

**Affiliations:** 1 Biochemistry and Molecular Biology Laboratory, Faculty of Pharmacy, MonastirTunisia; 2 Pulmonology Department, CHU Tahar Sfar, MahdiaTunisia; 3 Institute of Evolution Sciences, University of Montpellier-IIFrance; 4 Institute of Human Genetics, University of Montpellier-IIFrance

**Keywords:** alpha-1 antitrypsin, *SERPINA1 polymorphisms*, COPD, emphysema, lung function

## Abstract

Alpha-1-antitrypsin (AAT) plays an important role in the pathogenesis of emphysema, the pathological lesion underlying the majority of the manifestations of Chronic Obstructive Pulmonary Disease (COPD). In this study we tested the hypothesis that common AAT polymorphisms influence the risk of developing COPDs. We investigated PiM1 (Ala213Val), PiM2 (Arg101His), PiM3 (Glu376Asp), PiS (Glu264Val) and PiZ (Glu342Lys) *SERPINA1* alleles in 100 COPD patients and 200 healthy controls. No significant differences were observed in allele frequencies between COPD patients and controls, neither did haplotype analysis show significant differences between the two groups. A cross-sectional study revealed no significant relationship between common *SERPINA1* polymorphisms (PiM1, PiM2, PiM3) and the emphysematous type of COPD. In addition, FEV_1_ annual decline, determined during a two-year follow up period, revealed no difference among carriers of the tested polymorphisms.

Chronic obstructive pulmonary disease (COPD), a heterogeneous disorder, is a major cause of respiratory disability and the fourth major cause of death world-wide (World Health Organization, 2000). Exposure to cigarette smoke is recognized as the main environmental risk factor involved (Teramoto, 2007). Severe Alpha-1-antitrypsin deficiency (AATD) is a proven genetic risk factor, with about 80% of the subjects develop the disease between 30 to 40 years or earlier, in spite of only 1%-3% of COPD cases being due to severe AAT deficiency (Lomas and Silverman, 2001).

Alpha-1 antitrypsin (AAT) is a 52 kDa protein synthesized primarily by hepatocytes. Its main function is to inhibit the activity of neutrophile elastase in the lung. This is a protease capable of destroying the major structural proteins of the alveolar wall. Plasma AAT deficiency results in accelerated elastin degradation, leading to a loss of ventilator function and the subsequent development of emphysema (Mahadeva and Lomas, 1998). The AAT coding gene *SERPINA1* is highly polymorphic, with more than 125 SNPs reported in public databases, its most common alleles being the normal M alleles and its subtypes (PiM1Ala, PiM1Val, PiM2, PiM3), besides the deficient alleles PiS and PiZ (Crystal, 1990). About 95% of the individuals with severe AATD are homozygous for PiZt (ATS/ERS, 2003), whereas PiSZ heterozygotes have approximately one-third of the normal AAT serum level, besides being highly prone to the development of diseases (Dahl *et al.*, 2005). COPD risk among PiMZ heterozygotes has been previously analyzed with controversial results. Meta-analysis indicates a slight increase in risk of COPD in PiMZ individuals, with no significant lung-function impairment, compared to PiMM (Hersh *et al.*, 2004).

A few studies on *SERPINA1* common variants in COPD have been reported, also with controversial results. Matsuse *et al.* (1995), Shim (2001) and Kim *et al.* (2005) did not find a significant association between PiM1, PiM2 or PiM3 alleles and COPD, whereas Kwok *et al.* (2004) came across a significant increase in PiM1M3, PiM2M3 phenotypes and Gupta *et al.* (2005) reported a significant increase for the PiM3 allele in COPD patients.

For a better understanding of the association between *SERPINA1* polymorphisms and COPD risk, we designed a case-control study to detect differences in the frequencies of common SNP alleles, haplotypes and genotypes, between patients and controls in relation to common polymorphisms.

We also investigated the relationship between common *SERPINA1* polymorphisms and main COPD clinical manifestations. The major AAT neutrophile elastase inhibitory role is observed in alveolar parenchyma, the subsequent deficiency in plasma AAT concentration mainly resulting in emphysema (Needham and Stockley, 2004). We therefore compared the distribution of *SERPINA1* polymorphisms between the bronchial and emphysematous types of COPD. In addition, COPD patients underwent a two-year follow up, in order to evaluate the annual FEV_1_ (Forced Expiratory Volume in 1 s) decline rate in relation to common *SERPINA1* alleles. FEV_1_ is the hallmark of COPD since it is affected by inflammation and remodeling of the small airways as well as by emphysematous destruction of the terminal airspaces (Weiss *et al.*, 2003).

The study population consisted of 100 COPD subjects who attended the Pneumology Department of Tahar Sfar Hospital in Mahdia. Inclusion criteria for patients with COPD were as follows: FEV_1_ < 80% of predicted value adjusted for age, weight and height, and an improvement in FEV_1_ following bronchodilator inhalation < 12% of baseline FEV_1_. Asthmatic patients showing a persistent airflow obstruction were excluded. COPD phenotype identification was based on chest radiographic and high-resolution computerized tomography (HRCT) density findings. Clinical characteristics of COPD patients are summarized in Table S1. Follow-up examinations were conducted with patients over a two-year period after baseline, with annually repeated spirometry tests. AATD individuals were excluded from analysis. Two hundred healthy controls were enrolled for the case-control study. They were recruited from a blood donor's cohort of Fattouma Bourguiba Hospital in Monastir. Subjects with respiratory diseases, or any family history of lung disease, were excluded. The distribution of both patients and controls, according to demographic characteristics, is shown in Table S2. Prior written informed consent was obtained from all the subjects according to the research protocol approved by the local ethics committee.

Total genomic DNA was extracted from peripheral blood leucocytes by a phenol-chloroform method. PCR-RFLP was employed for genotyping of PiM1 (Ala213 GCG → Val GTG), PiS (Glu264 GAA → Val GTA) and PiZ (Glu342 GAG → Lys AAG) polymorphisms, as previously described (Ferrarotti *et al.*, 2004). Briefly, we performed PCR amplification using exon III primers to detect the S and 213Ala/Val variants and exon V for the Z variant. The reactions were carried out in an I-cycler Thermal Cycler (Bio-Rad Laboratories). 4 U of *SexAI* and *Hpy99I* restriction enzymes (New England Biolabs) were used to digest 4 μL each of exon III and exon V amplified DNA, respectively. Genotyping of PiM2 (Arg101 CGT → His CAT) and PiM3 (Glu376 GAA → Asp GAC) was performed using hybridization probe analysis on Light Cycler 480 Roche apparatus (Roche Diagnostics), using a commercial real-time assay (LightMix^®^, Roche Diagnostics). For each SNP, the primers flanking the SNP and the oligonucleotide probes were designed and synthesized by the manufacturer. The reaction mixture was prepared in a 96-well PCR plate and processed according to manufacturer's instructions. Real-time PCR cycling conditions were as follows: 95 °C for 5 min, followed by 35 cycles of 95 °C for 10 s, 62 °C for 15 s and 72 °C for 15 s. After amplification, PCR products were analyzed in a melting step of 40-95 °C. Melting data were analyzed using the Genescanning module of the LightCycler 480 software.

Annual FEV1 decline (ml/year) was calculated as the difference between follow-up and baseline observed FEV1 values, divided by the number of months between the two surveys, and multiplied by 12. SPSS v.10.0 software was used for statistical analysis. Categorical variables were presented as percentages, and intergroup differences were compared using χ^2^ test or Fisher's exact tests. Continuous variables, described as mean ± standard deviation, were compared between the groups using Student's t test. Hardy Weinberg equilibrium tests and the estimation of allele and haplotype frequencies were performed using HPlus v. 2.5 software.

The frequency of PiM1, PiM2, PiM3, PiS and PiZ alleles and genotypes between COPD patients and healthy controls, was determined and compared (Table 1). Genotypes for all the polymorphisms were within Hardy-Weinberg proportions. There was no significant difference in the genotypic and allelic distribution of normal PiM1, PiM2 and PiM3 variants between subjects and controls. Deficient PiS and PiZ alleles were only reported in patients, with no apparent significant difference in relation to controls. Ten haplotypes were selected for studying by the expectation maximization procedure (Table 2), with no significant differences being detected between patients and controls by statistical comparison.

COPD patients were classified according to their predominant phenotype as follows: 53 subjects showed the bronchial type of the disease (chronic bronchitis group; mean age: 72.1 ± 7.6 years); 47 presented a predominant parenchymal destructive change (centrolobular and panlobular emphysema groups; mean age: 69.5 ± 12.2 years). Univariate analysis was employed to verify whether there were differences in COPD phenotypes among *SERPINA1* genotypes (M1Ala containing *vs.* non M1Ala containing; M2 containing *vs.* non M2 containing and M3 containing *vs.* non M3 containing). The relationship between FEV_1_ annual decline, smocking and BMI and COPD phenotypes was also examined. No significant differences were detected (Table 3).

Lung function impairment in patients was assessed by the annual FEV_1_ decline rate. After exclusion of AAT deficient individuals, 96 patients underwent a two-year follow up Annual FEV_1_ decline means were compared according to age, smoking habits and *SERPINA1* polymorphisms. No significant relationship between annual FEV_1_ decline and the tested variables was detected (Table 4).

In summary, our findings are consistent with observations that there is no significant difference in the frequency of common *SERPINA1* variants in COPD patients, when compared to healthy controls. We also verified that there is no correlation between these alleles and the manifestation of emphysema, since there was no difference in their distribution in patients with bronchial and emphysematous types of COPD. Furthermore, no significant relationship between *SERPINA1* polymorphisms and annual FEV_1_ decline evaluated over a two-year period in COPD patients was found. To our knowledge, this is the first report on clinical manifestations of COPD in relation to common AAT variants.

## Supplementary Material

The following online material is available for this article:

Table S1 Clinical characteristics of COPD patients at baseline.

Table S2 Demographic characteristics of COPD patients and healthy controls

This material is made available as part of the on-line article from http://www.scielo.br.gmb.

## Figures and Tables

**Table 1:** *SERPINA1*genotypes and alleles in COPD patients and controls.

	Genotype				Allele		HWE p
Ala213Val	CC	CT	TT		C	T	
Controls	0.63	0.33	0.04		0.79	0.21	0.699^a^
Patients	0.63	0.31	0.06		0.78	0.22	0.418^a^
p	0.764^a^	0.887^a^	0.516^a^			0.671^a^	
OR (95% CI)						1.096 (0.718-1.672)	
Arg101His	GG	GA	AA		G	A	
Controls	0.57	0.38	0.05		0.77	0.23	0.440^a^
Patients	0.61	0.36	0.03		0.78	0.22	0.380^a^
p	0.862^a^	0.937^a^	0.477^b^			0.770^a^	
OR (95% CI)						0.939 (0.617-1.430)	
Glu376Asp	AA	AC	CC		A	C	
Controls	0.49	0.45	0.06		0.73	0.28	0.217^a^
Patients	0.53	0.39	0.08		0.73	0.27	0.648^a^
p	0.348^a^	0.413^a^	0.497^a^			0.798^a^	
OR (95% CI)						0.951 (0.645-1.402)	
Glu264Val	AA	AT	TT		A	T	
Controls	1	0	0		1	0	N/A
Patients	0.98	0.02	0		0.99	0.01	0.917^a^
p	0.193^b^	0.193^b^	N/A			0.193^b^	
OR (95% CI)						10.199 (0.487-213.501)	
Glu342Lys	GG	GA	AA		G	A	
Controls	1	0	0		1	0	N/A
Patients	0.98	0.02	0		0.99	0.01	0.917^a^
p	0.193^b^	0.193^b^	N/A			0.193^b^	
OR (95% CI)						10.199 (0.487-213.501)	

HWE: Hardy-Weinberg Equilibrium.^a^Pearson's χ^2^ test; ^b^Fisher's exact test.

**Table 2:** *SERPINA1* SNP haplotypes in COPD patients and controls.

Haplotype^a^	Patients	Controls	p^b^	OR	95% CI
GTAGA	0.48265	0.48773	1	1	N/A
GCAGA	0.21581	0.20572	0.864	1.04	0.67-1.61
ATAGC	0.18798	0.20385	0.683	0.91	0.56-1.46
GTAGC	0.08046	0.07999	0.974	0.99	0.50-1.96
ATAGA	0.01730	0.02270	0.663	0.77	0.24-2.50
ATTGA	0.00526	0.00000	N/A	N/A	N/A
ATAAC	0.00524	0.00000	N/A	N/A	N/A
GCAAA	0.00524	0.00000	N/A	N/A	N/A
GTAAA	0.00005	0.00000	N/A	N/A	N/A
ACAGC	0.00001	0.00000	N/A	N/A	N/A

^a^Haplotype frequency determined using expectation maximization method; ^b^Fisher's exact test.

**Table 3:** Association studies of bronchial and emphysematous COPD types with annual FEV1 decline (ΔFEV_1_), cigarette smoking, body mass index (BMI) and *SERPINA1* genotypes.

Type/Class	COPD phenotype		p
	CB	CLE+PEL	
ΔFEV_1_ (ml/year)	171 ± 137	232 ± 239	0.243^a^
Cumulative cigarette consumption	59.41 ± 29.22	51.97 ± 28.68	0.230^a^
BMI (kg/m^2^)	24.33 ± 3.70	22.77 ± 5.21	0.117^a^
M1Ala	0.41	0.53	
No M1Ala	0.58	0.47	0.306^b^
M2	0.45	0.45	
No M2	0.54	0.54	1^b^
M3	0.39	0.53	
No M3	0.60	0.46	0.184^b^

CB: Chronic Bronchitis; CLE: Centrolobular Emphysema; PLE: Panlobular Emphysema; Cumulative cigarette consumption = number of packs smoked per day multiplied by years of consumption. M1Ala: heterozygous and homozygous for Ala213 allele; no M1Ala: homozygous for Val213 allele; M2: heterozygous and homozygous for His101allele; no M2: homozygous for Arg101 allele; M3: heterozygous and homozygous for Asp376 allele; no M3: homozygous for Glu376 allele. ^a^Student's t test; ^b^Pearson's χ^2^ test.

**Table 4:** Association studies of lung function impairment with smoking, body mass index (BMI), age and *SERPINA1* genotypes.

Type/class	ΔFEV_1_ (mL/year)	p^a^
Smoking status		
Never	151 ± 131	
Smoker	197 ± 191	0.683
Cumulative cigarette consumption		
< mean	164 ± 179	
> mean	230 ± 194	0.197
BMI (kg/m^2^)		
< mean BMI	221 ± 239	
> mean BMI	178 ± 144	0.408
Age		
< mean age	165 ± 109	
> mean age	207 ± 211	0.455
M1Ala	195 ± 177	
No M1Ala	194 ± 205	0.983^a^
M2	195 ± 156	
No M2	195 ± 204	0.999
M3	205 ± 212	
No M3	187 ± 152	0.726

Cumulative cigarette consumption = number of packs smoked per day multiplied by years of consumption. M1Ala: heterozygous and homozygous for Ala213 allele; no M1Ala: homozygous for Val213 allele; M2: heterozygous and homozygous for His101 allele; no M2: homozygous for Arg101 allele; M3: heterozygous and homozygous for Asp376 allele; no M3: homozygous for Glu376 allele. ^a^Student's t test.
