# Supplementary material for: Alpha-1 antitrypsin gene polymorphism in Chronic Obstructive Pulmonary Disease (COPD)
Source: Genet Mol Biol. 2010 Mar 1;33(1):23–6. doi: 10.1590/S1415-47572009005000107 (PMC3036098; doi:10.1590/S1415-47572009005000107)
Supplement: Table S1 — Clinical characteristics of COPD patients at baseline. [file gmb-33-1-23-suppl1.pdf]

Table S1 - Clinical characteristics of COPD patients at baseline.

Clinical characteristics	COPD Patients (n = 100)
COPD phenotype	
CB	52%
CB+CLE	35%
PLE	13%
Spirometry	
FEV_1	1.16 ± 0.61
FEV_1 % pred	41.7 ± 16.8
FVC	1.55 ± 0.78
FVC % pred	43.7 ± 18.5
RV %	2.7 ± 3.4
Blood gases	
PaO_2	69.2 ± 10.6
$PaCO_2$	43.2 ± 10
SaO_2	93 ± 3.8
COPD stage	
I	2%
II	24%
III	48%
IV	26%

CB: Chronic Bronchitis; CLE: Centrolobular Emphysema; PLE: Panlobular Emphysema; FEV_1: Forced Expiratory Volume in 1 s (L); FVC: Forced Vital Capacity (L); FEV_1 % pred: percentage of the predicted FEV_1 value adjusted to age, height and weight. FVC % pred: percentage of the forecast FVC value adjusted to age, height and weight. RV: Reversibility of baseline FEV_1 following bronchodilator inhalation; PaO2: arterial oxygen partial pressure (mm Hg); $PaCO_2$: arterial carbon monoxide partial pressure (mm Hg); SaO_2: Arterial oxygen saturation (%).
