# Supplementary material for: Alpha-1 antitrypsin gene polymorphism in Chronic Obstructive Pulmonary Disease (COPD)
Source: Genet Mol Biol. 2010 Mar 1;33(1):23–6. doi: 10.1590/S1415-47572009005000107 (PMC3036098; doi:10.1590/S1415-47572009005000107)
Supplement: Table S2 — Demographic characteristics of COPD patients and healthy controls [file gmb-33-1-23-suppl2.pdf]

Table S2 - Demographic characteristics of COPD patients and healthy controls.

Demographic characteristics	COPD patient (n = 100)	Controls (n = 200)	p
Age	69.45 ± 12.34	43.86 ± 8.97	0.00[a]
male:female	98:2	189:11	0.15[b]
BMI	24.5 ± 4.0	24.4 ± 2.9	0.80[a]
Smoking status			
Never	7	16	
Ex	52	89	
Current	41	95	0.09[b]
Cumulative cigarette consumption	53.74 ± 29.76	48.41 ± 23.86	0.14[a]
Geographic origin in Tunisia			
North	0	4	
Center	100	192	
South	0	4	0.08[b]

BMI: Body mass index, weight (kg)/(height (m))^2. Cumulative cigarette consumption = number of packs smoked per day multiplied by years of consumption.

[a]Student's t test; [b]Pearson's χ^2 test.
